# Respiratory symptoms are poor predictors of concomitant chronic obstructive pulmonary disease in patients with primary Sjögren’s syndrome

**DOI:** 10.1007/s00296-017-3678-5

**Published:** 2017-02-27

**Authors:** Victor Strevens Bolmgren, Peter Olsson, Per Wollmer, Roger Hesselstrand, Thomas Mandl

**Affiliations:** 1 Department of Clinical Sciences Malmö, Lund University, Malmö, Sweden; 2 Department of Translational Medicine, Lund University, Malmö, Sweden; 3 Department of Clinical Sciences Lund, Lund University, Lund, Sweden; 4 Department of Rheumatology, Skåne University Hospital, IM Nilssons gata 32, 205 02 Malmö, Sweden; 5 Department of Clinical Physiology, Skåne University Hospital, Malmö, Sweden; 6 Department of Rheumatology, Skåne University Hospital, Lund, Sweden

**Keywords:** Sjögren’s syndrome, Pulmonary disease, Co-morbidities, Imaging

## Abstract

Involvement of the respiratory system, in particular dry airways and chronic obstructive pulmonary disease (COPD), is common in patients with primary Sjögren’s syndrome (pSS). As respiratory symptoms are also common in pSS patients and may have different etiologies, we wanted to evaluate the amount and impact of respiratory symptoms in out-patients with pSS and to assess if such symptoms are related to concomitant COPD. The St George’s Respiratory Questionnaire (SGRQ) was used to assess respiratory symptoms. SGRQ scores were compared between 51 consecutive pSS patients, in an out-patient setting, and 80 population-based controls. The patients were also studied by pulmonary function tests and CT scans of the lungs to assess signs of obstructive airway disease, including COPD, as well as to assess signs of interstitial lung disease (ILD). 41 and 18% of pSS patients were found to have COPD and radiographic signs of ILD, respectively. pSS patients had significantly higher SGRQ scores compared to controls, but no significant differences in SGRQ scores were found between patients with and without COPD. Neither did the small group of pSS patients with ILD significantly differ in SGRQ scores in comparison to patients without ILD. Respiratory symptoms were common in pSS, but were not more common in patients with concomitant COPD. Since pulmonary involvement in pSS is associated with an increased mortality and respiratory symptoms is a poor marker for pulmonary involvement, we suggest that pulmonary function tests should be performed liberally in all pSS patients regardless of symptoms.

## Introduction

Primary Sjögren’s syndrome (pSS) is a chronic autoimmune disease predominantly affecting exocrine glands resulting in diminished secretion and subsequent sicca symptoms [1, 2]. Extraglandular manifestations affecting various organs including the lungs are common amongst pSS patients [3–9] and approximately 50% of pSS patients report dry cough [3]. However, involvement of the respiratory system in pSS entails not only dry airways, but also obstructive airway disease [8], including chronic obstructive pulmonary disease (COPD) [5, 6], as well as interstitial lung disease (ILD) [3, 4, 10], all of which can co-exist in the same patient. Furthermore, all types of respiratory involvement can result in respiratory symptoms. Although pulmonary involvement seems to be subclinical in a substantial fraction of pSS patients [4], concomitant pulmonary involvement in pSS has been shown to be associated with a fourfold increased risk of mortality after 10 years of disease [7]. It has previously been shown that COPD is a common finding in pSS patients, even among never-smoking patients [5, 6]. To the best of our knowledge, no previous study has investigated to what degree COPD contributes to the respiratory symptoms in pSS patients. Since concomitant lung disease is associated with a poorer survival in pSS patients, it is important to determine if evaluation of pulmonary involvement, as evaluated by pulmonary function tests (PFTs) and radiographic evaluations of the lungs, to diagnose concomitant COPD and ILD, should be performed mainly in pSS patients with symptoms or if these should be performed regardless of symptoms. Therefore, the aims of this study were (1) to compare respiratory symptoms in pSS patients with population-based controls and (2) to study whether such symptoms are associated with concomitant COPD, as this is a common finding in pSS patients in general.

## Materials and methods

At the Department of Rheumatology, Skåne University Hospital Malmö, Sweden, consecutive pSS patients have been registered and clinically followed-up since 1984. Since our department is the only unit in this area following pSS patients, they represent a rather unselected spectrum of pSS patients. Fifty-one consecutive outpatients diagnosed with pSS, according to the American-European Consensus Group (AECG) criteria [11], seen from May to December in 2012, at our department were included in the study. These patients were in a previous study investigated for concomitant pulmonary involvement by PFTs, CT scans of the lungs and the St George’s Respiratory Questionnaire (SGRQ) on respiratory symptoms [12, 13]. Briefly, PFT included static and dynamic spirometry, from which the vital capacity (VC), total lung capacity, residual volume, forced expiratory volume in 1 s (FEV_1_), and FEV_1_/VC ratio, could be calculated. Diffusion capacity for carbon monoxide (*D*_L CO_) was measured by the single-breath technique. FEV_1_ and VC were measured before and after 1.0 mg of inhaled terbutaline and VC after reversibility test and FEV_1_ reversibility was calculated. Radiographic features of the lungs were evaluated by conventional CT of the chest. Because signs of ILD as opposed to signs of obstructive pulmonary disease are less commonly detected in the general pSS population, we mainly wanted to evaluate the latter in this study. Also due to radiation hygienic reasons, conventional CT was chosen instead of HRCT. The presence of a reticular pattern, ground glass attenuation, honeycombing, central as well as traction bronchiectasis, emphysema, pulmonary cysts, and air trapping was noted. COPD was defined according to the Global Initiative for Chronic Obstructive Lung disease (GOLD) criteria [14] and ILD, according to the presence of ground glass opacities, honeycombing or peripheral traction bronchiectasis in CT scans of the lungs. Further characteristics of the patients are shown in Table 1. 80 population based controls (median age 61, range 31–80 years, 43 females), living in the county of Skåne, and denying having been diagnosed with a rheumatologic disease, asthma or COPD as well as using bronchodilators and/or inhalation steroids the last 6 months, were randomly selected from the Swedish population registry and included in the study. 49, 44 and 8% of the controls were never-smokers, prior smokers, and current smokers, respectively. A brief questionnaire on smoking habits and information on age, gender, height and weight as well as the SGRQ were mailed by post to the controls.

**Table 1:** Demographic and clinical characteristics of the patients with primary Sjögren’s syndrome included in the study

	Patients (*n* = 51)
Age (years)	60 ± 12
Female patients *N* (%)	49 (96)
Disease duration (years)	17 ± 12
Current/prior/never smokers *N* (%)	4/20/27 (8/39/53)
Anti-SS-A antibody seropositives *N* (%)	40 (78)
Anti-SS-B antibody seropositives *N* (%)	24 (47)
ANA seropositives *N* (%)	40 (78)
RF seropositives *N* (%)	26 (51)
IgG (g/L)	13.8 ± 5.1
C3 (g/L)	1.03 ± 0.25
C4 (g/L)	0.18 ± 0.07
Focus score ≥1 (positive/negative/not assessed) *N* (%)	37/3/11 (73/6/22)
ESSDAI total score	7 ± 6
ESSPRI total score	6 ± 2
Fulfilling GOLD criteria for COPD *N* (%)	21 (41)
VC (L)	3.3 ± 0.8
FEV_1_ (L)	2.3 ± 0.6
FEV_1_/VC ratio	0.69 ± 0.08
*D*_L CO_ (mmol/min kPa)	6.5 ± 1.9
Radiographic signs of ILD of the below *N* (%)	9 (18)
Peripheral traction bronchiectasias *N* (%)	4 (8)
Ground glass opacities *N* (%)	6 (12)
Honey combing *N* (%)	0 (0)

Results are presented as mean ± SD or as absolute numbers (% with abnormal findings)

*GOLD* Global Initiative for Chronic Obstructive Lung Disease, *COPD* chronic obstructive pulmonary disease, *VC* vital capacity, *FEV*_*1*_ forced expiratory volume in 1 s, *D*_*L CO*_ diffusion capacity for carbon monoxide

All subjects had to fill out a structured questionnaire on smoking habits with queries on current and past smoking habits with mean cigarette smoking per day. Tobacco consumption, expressed as pack-years, could thus be calculated. Subjects were also asked to state their height and weight from which the body mass index (BMI) could be calculated. We used the Swedish version of the SGRQ to evaluate the respiratory symptoms and consequences of these [13]. The questionnaire generates four different scores: symptoms, activity, impact and a total score based on the former three. Scores range from 0 to 100 and the lower the score the better the health. The Swedish version has been tested for reliability and validity and compares well with the original. The questionnaire has previously been described in more detail [12, 13]. After scoring the questionnaires, the controls were stratified into females and males and a linear regression analysis was performed in which age, BMI and pack-years were added as co-variates to calculate gender, age, BMI and pack-year standardised values for the SGRQ subscores and total score. From the equations of these analyses, expected values for the SGRQ subscores and total score in the pSS patients were calculated. The expected SGRQ-scores, taking into account gender, age, tobacco consumption and BMI, were then compared with the actual SGRQ-scores found in the pSS patients. To compare the SGRQ scores in pSS patients with COPD (pSS+COPD) vs. without COPD (pSS–COPD) we calculated the deviation of SGRQ scores from expected values in each pSS patient and compared the groups. The same calculation was performed to compare pSS patients with (pSS+ILD) and without (pSS–ILD) radiographic signs of ILD.

## Statistics

To calculate the expected SGRQ scores for the pSS-patients a linear regression was performed. When comparing the expected SGRQ scores with the actual SGRQ scores in the pSS patients, a paired samples Student’s *t* test was used. When comparing the deviation in SGRQ-results from expected values, between pSS+COPD with pSS–COPD and pSS+ILD with pSS–ILD, respectively, the Mann–Whitney *U* test was used. Results were presented if not stated otherwise as median (interquartile range) and *p* values < 0.05 were considered statistically significant.

## Ethics

The study was approved by the local ethics board (Lund University 2015/310). All patients gave written informed consent and the study was performed according to the Declaration of Helsinki.

## Results

**Fig. 1:**
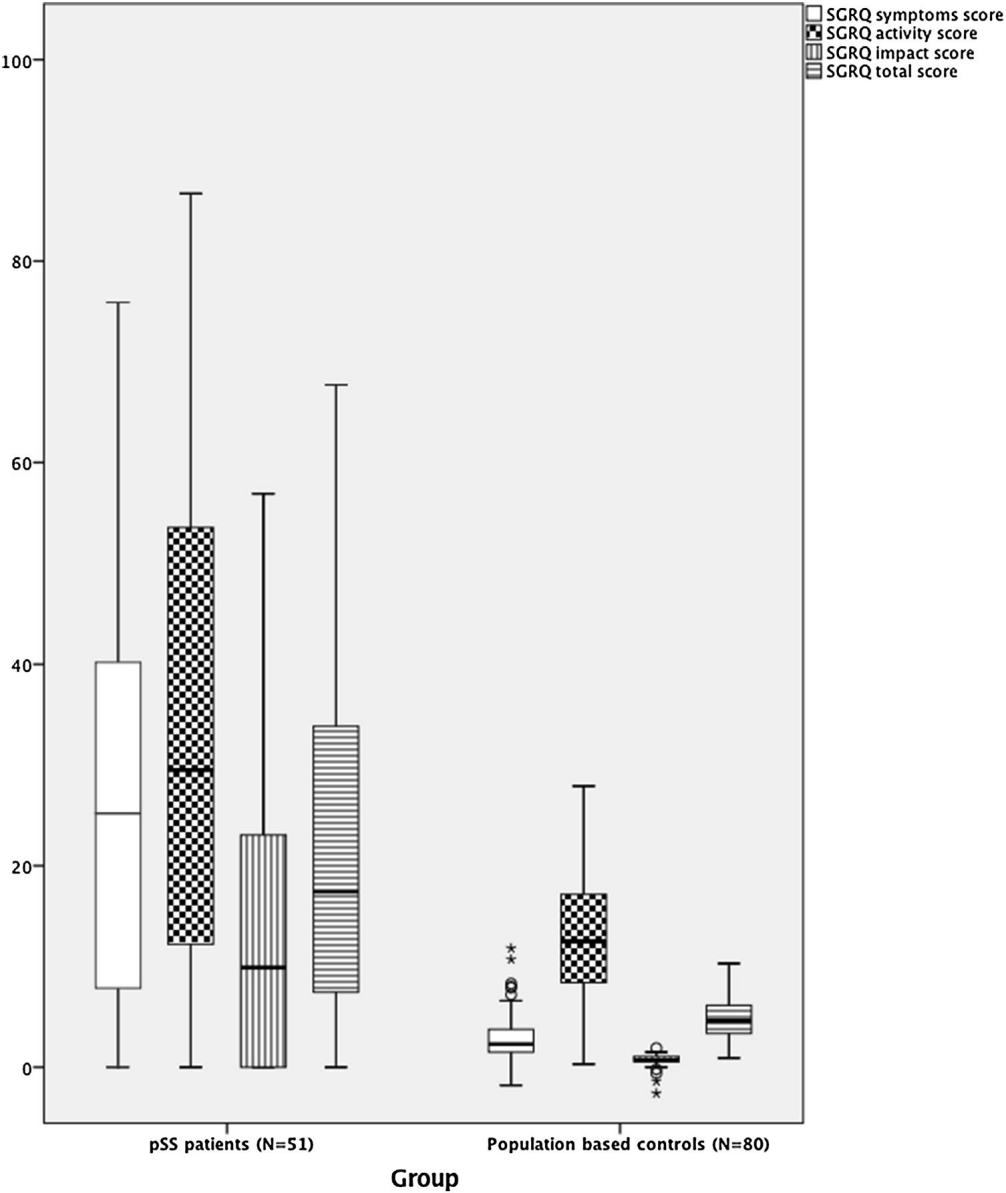
Results of the St George’s Respiratory Questionnaire (SGRQ) in pSS patients and expected values based on the results from population based controls. The *horizontal line* in the *middle of the box* represents the median value and the *height of the box* the interquartile range (IQR). The *horizontal bars* below and *above the box* define the range

41% of pSS patients fulfilled GOLD criteria for COPD and 18% showed radiographic signs of ILD. pSS patients had significantly increased SGRQ symptoms (*p* < 0.001), SGRQ activity (*p* < 0.001), SGRQ impact (*p* < 0.001) as well as SGRQ total scores (*p* < 0.001) in comparison to expected scores based on the results of the population based controls (Fig. 1). However, there was no significant difference in the deviation of SGRQ symptoms, SGRQ activity, SGRQ impact or SGRQ total scores from expected values in pSS patients with and without COPD (Table 2). In addition, there was no significant difference in the deviation of SGRQ symptoms, SGRQ activity, SGRQ impact or SGRQ total scores from expected values in pSS patients with and without radiographic signs of ILD (Table 2).

**Table 2:** Deviation of SGRQ (ΔSGRQ) subscores from expected values in pSS patients with COPD (pSS+COPD) vs. pSS patients without COPD (pSS–COPD) as well as in pSS patients with ILD (pSS+ILD) vs. pSS patients without ILD (pSS–ILD)

	pSS+COPD (*n* = 21)	pSS–COPD (*n* = 30)	*p* value
ΔSGRQ symptoms score	24.4 (6.5; 41.6)	21.3 (1.2; 36.7)	0.39
ΔSGRQ activity score	18.8 (6.3; 47.3)	21.8 (0.8; 35.6)	0.85
ΔSGRQ impact score	10.6 (0.1; 23.8)	6.6 (−0.7; 22.0)	0.42
ΔSGRQ total score	23.0 (2.5; 31.5)	18.5 (1.2; 27.4)	0.48

	pSS+ILD (*n* = 9)	pSS–ILD (*n* = 42)	*p* value
ΔSGRQ symptoms score	33.6 (0.3; 49.1)	22.3 (3.1; 36.7)	0.54
ΔSGRQ activity score	34.7 (8.7; 50.3)	15.4 (−2.6; 38.7)	0.24
ΔSGRQ impact score	12.0 (1.2; 22.9)	7.9 (−0.6; 23.0)	0.60
ΔSGRQ total score	27.4 (5.3; 31.6)	14.4 (1.9; 27.6)	0.26

Results are presented as deviation in points from expected values [median (IQR)]

## Discussion

In this study, we showed that respiratory symptoms were more common in pSS patients than in population-based controls, even after taking age, gender, tobacco consumption and BMI into account. Furthermore, these symptoms lead to restrictions in daily physical activities and had psychosocial consequences for the pSS patients. Although a large proportion of patients fulfilled GOLD criteria for COPD, concomitant COPD did not contribute significantly to the reported respiratory symptoms in these patients. Although the group of patients with concomitant ILD was too small to draw any firm conclusions, these patients did not significantly differ from patients without ILD with regard to respiratory symptoms either. Nevertheless, the results of the current study imply that airway sicca is the most likely cause of respiratory symptoms in unselected pSS patients. Another explanation could also be inflammatory processes in close proximity to the small airways resulting in bronchial hyperreactivity and respiratory symptoms [8, 15–17]. Since airway sicca, COPD and ILD may co-exist in the same patient, another explanation could also be that respiratory symptoms caused by COPD and ILD in pSS patients are obscured by concomitant superimposed symptoms due to airway sicca.

Early detection of pulmonary involvement seems important considering the increased mortality in pSS patients with pulmonary manifestations [7, 18, 19], particularly if improved disease-modifying treatments, in the future, gain a wider use in the treatment of pSS patients with systemic disease [4, 20]. Therefore, we suggest that patients with pSS should be evaluated by PFT, to screen for concomitant pulmonary involvement, both at baseline and liberally at follow-up, regardless of respiratory symptoms, since respiratory symptoms seem to be a poor marker for concomitant COPD in pSS patients.

The strengths of our study were the use of consecutive patients, diagnosed with pSS in accordance with the generally acknowledged AECG criteria and the use of population based controls. Limitations of the study include the limited size of the study with patients having relatively mild pulmonary involvement, considering the fact that none of the patients had radiographic signs of more severe ILD. Therefore, patients with more severe ILD may have more pronounced symptoms that the SGRQ would detect. Still, a substantial fraction of patients fulfilled GOLD criteria for COPD, and the symptoms due to COPD could not be detected by the SGRQ in pSS patients although the SGRQ has previously been validated in ordinary COPD patients [13]. Secondly, the pSS patients in this study were, due to radiation hygienic purposes, only evaluated by conventional and not high resolution CT (HRCT) scans, and therefore less likely to be diagnosed with concomitant ILD. However, according to a previous study, using HRCT in evaluation of pulmonary involvement in pSS patients, HRCT signs of ILD were quite rare in comparison with spirometric signs of COPD, in pSS patients in general [5]. Taken together, this implies that the association between respiratory symptoms and ILD has to be further studied in patients with more severe ILD. Thirdly, inclusion of a second positive control group consisting of COPD patients in general would have strengthened the findings of this study. Unfortunately, no second control group was studied. Finally, the presence of concomitant undiagnosed COPD, asthma and congestive heart disease, all possibly resulting in respiratory symptoms, was not ruled out amongst controls.

In conclusion, we showed that respiratory symptoms were more common amongst pSS patients than in the normal population. Concomitant COPD did not seem to significantly contribute to these, suggesting that other factors are more important in eliciting such symptoms in pSS patients, of which airway sicca is probably the most important. Since pulmonary involvement in pSS is associated with an increased mortality and respiratory symptoms is a poor marker for pulmonary involvement, we suggest that PFT should be performed liberally in all pSS patients regardless of symptoms to enable early detection of pulmonary involvement in these patients.
